# Language prediction mechanisms in human auditory cortex

**DOI:** 10.1038/s41467-020-19010-6

**Published:** 2020-10-16

**Authors:** K. J. Forseth, G. Hickok, P. S. Rollo, N. Tandon

**Affiliations:** 1 Vivian L. Smith Department of Neurosurgery, McGovern Medical School, Houston, TX USA; 2 Department of Cognitive Sciences, University of California, Irvine, CA USA; 3 Memorial Hermann Hospital, Texas Medical Center, Houston, TX USA

**Keywords:** Cortex, Language, Neural encoding

## Abstract

Spoken language, both perception and production, is thought to be facilitated by an ensemble of predictive mechanisms. We obtain intracranial recordings in 37 patients using depth probes implanted along the anteroposterior extent of the supratemporal plane during rhythm listening, speech perception, and speech production. These reveal two predictive mechanisms in early auditory cortex with distinct anatomical and functional characteristics. The first, localized to bilateral Heschl’s gyri and indexed by low-frequency phase, predicts the timing of acoustic events. The second, localized to planum temporale only in language-dominant cortex and indexed by high-gamma power, shows a transient response to acoustic stimuli that is uniquely suppressed during speech production. Chronometric stimulation of Heschl’s gyrus selectively disrupts speech perception, while stimulation of planum temporale selectively disrupts speech production. This work illuminates the fundamental acoustic infrastructure—both architecture and function—for spoken language, grounding cognitive models of speech perception and production in human neurobiology.

## Abstract (web summary)

The human brain fluently parses continuous speech during perception and production. Using direct brain recordings coupled with stimulation, the authors identify separable substrates underlying two distinct predictive mechanisms of “when” in Heschl’s gyrus and “what” in planum temporale.

## Introduction

Humans efficiently extract speech information from noisy acoustic signals and segment this into meaningful linguistic units. This complex and poorly understood process is fluidly accomplished for a wide range of voices, accents, and speaking rates^1^. Given the quasi-periodic and hierarchical structure of speech^2^, the computational load associated with its decoding can be reduced by utilizing temporal prediction^3^. Anticipating the arrival of salient acoustic information could enable optimal potentiation of neural networks^4^ and discretization of the continuous signal into linguistic elements^5–7^. This perspective, the active sensing framework^8^, anticipates interactions between bottom-up sensory input and top-down predictive modulation of neuronal dynamics. Evidence for cortical entrainment—the synchronization of extrinsic quasi-periodic stimuli and intrinsic neural activity—in the auditory domain^9^ and during speech perception^10–14^ has driven speculation that cortical oscillations may enable temporal prediction. In addition, speech production is also thought to rely upon predictive mechanisms. Several prominent models require that the brain anticipate the sensory consequences of speech^15,16^ and this central tenet has been buttressed by strong evidence^17–19^. It remains unclear, however, which levels of auditory cortical processing are involved in this process and where such mechanisms are instantiated in the cortex.

We elucidate the mechanisms by which auditory cortex anticipates rhythms and, further, whether such mechanisms may extend to optimize the processing of quasi-rhythmic acoustic input during language perception. To investigate the neurobiology of prediction in early auditory cortex, we use two tasks: amplitude-modulated white noise and spoken naming to definition. The white noise stimulus comprises a rhythmic pattern followed by a constant-amplitude interval; patients are tasked with detecting the occurrence of a peri-threshold tone in the latter interval. Neural encodings of prediction should uniquely persist during the latter period, while other cortical signals—including evoked response potentials and envelope tracking—would be limited to the rhythmic period. Next, we examine the cortical response to natural language speech for these same encoding signatures. Finally, to reveal causal involvement of specific neuroanatomic substrates, we apply chronometric stimulation to targeted structures during naming.

**Fig. 1:**
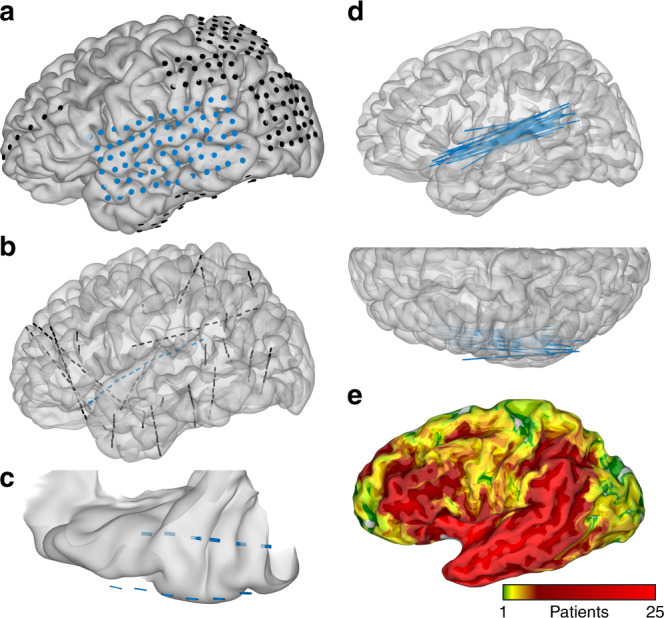
Supratemporal depth probe trajectory and complete coverage map. Electrodes represented relative to cortical models. **a** Grid electrodes localized relative to patient-specific cortical model, including a lateral temporal grid. **b** Same patient with a second implant using stereotactic depth electrodes, including an anteroposterior supratemporal trajectory. **c** Dorsal view of the supratemporal plane in the same patient with depth electrodes in Heschl’s gyrus and planum temporale, as well as the closest grid electrodes over lateral superior temporal gyrus. **d** All anteroposterior supratemporal trajectories superimposed in Talairach space relative to the MNI27 atlas. **e** Recording zone density from all electrodes (37 patients, 838 grid electrodes, 6669 depth electrodes) on an inflated MNI27 atlas.

The characterization and localization of predictive mechanisms for language function requires a methodology with high temporal resolution, fine spatial resolution, and direct access to neuronal populations in human early auditory cortex. We use large-scale intracranial recordings (37 patients), focusing on depth electrodes placed along the anteroposterior extent of the supratemporal plane (Fig. 1). This innovative surgical approach enables simultaneous recordings from planum polare, Heschl’s gyrus, and planum temporale. These experiments yield crucial insights into the rapid, transient dynamics of predictive timing and predictive coding—prediction of when and what^20^—in Heschl’s gyrus and planum temporale.

## Results

### Cortical response to low-level acoustic stimuli

**Fig. 2:**
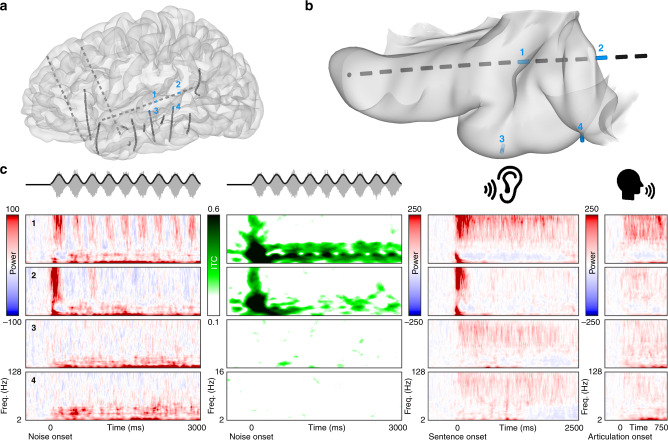
Cortical response to white noise and natural spoken language. Cortical responses in a single patient to low- and high-level auditory stimuli. **a** Location of all electrodes implanted in this patient relative to a model of the pial surface. **b** The supratemporal plane, isolated from the cortical model and viewed from above. All electrodes along the supratemporal depth probe are shown, as well as the two most superficial electrodes from probes in lateral superior temporal gyrus. Four electrodes are highlighted in blue with labels. **c** Neural responses at the four highlighted electrodes: (1) HG/TTS, (2) PT, (3), mediolateral superior temporal gyrus, and (4) posterolateral superior temporal gyrus. The first two columns show the spectral decomposition of the power and phase response, respectively, during white noise listening. The latter two columns show the power response during natural language listening and production, respectively.

**Fig. 3:**
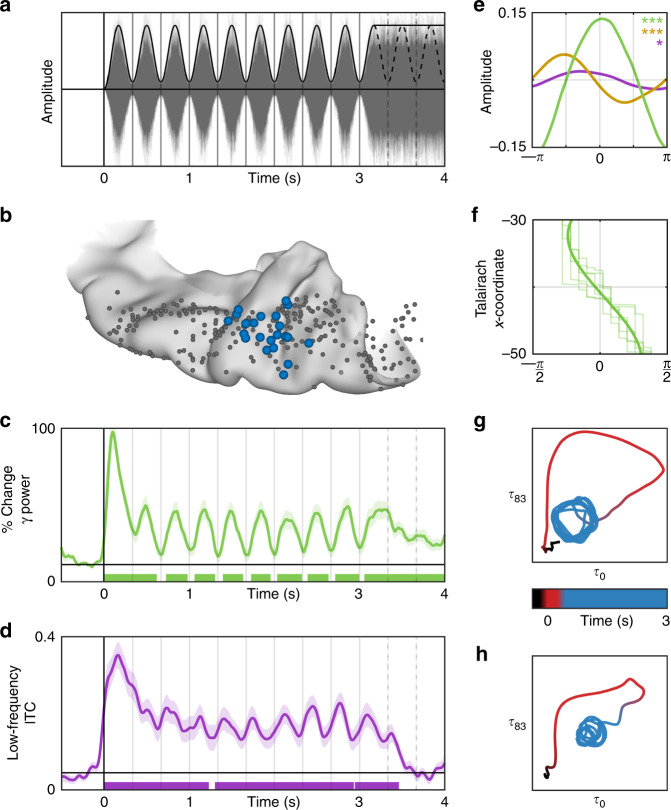
Response of Heschl’s gyrus to rhythmic white noise. Cortical response to rhythmic white noise. **a** 3 Hz amplitude-modulated white noise stimulus. **b** The most active electrode (blue) was selected from all electrodes (gray) in each patient with a supratemporal depth probe (*n* = 26). These were used for the following analyses. **c** Average percent change in high-gamma (65–115 Hz) power relative to pre-stimulus baseline. Shaded area represents ±1 standard error of the mean. Significance was determined with the Wilcoxon signed-rank test at an alpha level of *p* < 0.01 using familywise error correction. **d** Average absolute change in low-frequency (2–15 Hz) inter-trial coherence (ITC) from a pre-stimulus baseline. **e** Average amplitude of low (purple), beta (yellow), and high-gamma (green) frequencies relative to the stimulus phase during the sustained response (pulses 2–9) demonstrating frequency-multiplexed encoding of acoustic envelope. Significance (*p < 0.05, **p < 0.01, ***p < 0.001) was determined with bootstrapping (*n* = 1000) of Kullback–Leibler divergence from a uniform distribution across phase for low (*p* = 0.038), beta (*p* < 0.001), and high-gamma (*p* < 0.001) frequencies. **f** Spatial distribution of peak high-gamma power timing relative to stimulus phase demonstrates a traveling wave (velocity 0.1 m/s) that begins medially at the insular boundary (top) and progresses to the lateral edge (bottom). The mean wave (dark lines) is superimposed over the wave at each pulse (light lines). **g**, **h** Phase space trajectory at a quarter period delay (83 ms) in high-gamma power (top) and low frequency ITC (bottom). Time indicated by color: pre-stimulus baseline (black), red (first acoustic pulse), blue (pulses 2–9).

We observed a sustained multispectral response of early auditory cortex during rhythmic amplitude-modulated white noise (80% depth at 3 Hz for 3 s, then constant amplitude for 1 s). Heschl’s gyrus and the transverse temporal sulcus (HG/TTS; Fig. 2a, b, Electrode 1) encoded stimulus features in high-frequency power and low-frequency phase (Fig. 2c). These results were robust across the patient cohort, both in high-gamma power (Fig. 3c) and in low-frequency phase (Fig. 3d). Following a low-latency high-magnitude broadband response to stimulus onset, HG/TTS exhibited a sustained response to subsequent acoustic pulses. Phase space trajectories of high-gamma power (Fig. 3g) and low-frequency phase (Fig. 3h) revealed three clearly dissociable states corresponding to rest (pre-stimulus), stimulus onset, and sustained activity (beginning with the second pulse). Electrodes in lateral superior temporal gyrus (Fig. 2a, b, Electrodes 3 and 4 and Supplementary Fig. 1a) showed no evidence of a sustained response to the white noise stimulus (Fig. 2c and Supplementary Fig. 1c). In contrast, a sustained response was recorded in all patients with a supratemporal depth probe in the language-dominant hemisphere (*n* = 22). Patients with homologous electrodes in the language non-dominant hemisphere (*n* = 5) demonstrated an equivalent sustained response. This was also observed for faster modulations of the temporal envelope (5 and 7 Hz, Supplementary Fig. 2).

High-gamma, beta, and low-frequency power together yielded a frequency-multiplexed encoding of acoustic envelope (Fig. 3e). High-gamma power was in-phase with the stimulus, beta power was resynchronized at the trough of the stimulus, and low-frequency power was modulated by the rising slope of each pulse—the acoustic edge. These distinct and asymmetric bandlimited responses may represent separable cortical processes each engaged by the stimulus^21^. Low-frequency phase was reset at the acoustic edge. Spectral decomposition of the low-frequency phase response (Fig. 2c and Supplementary Fig. 1b) demonstrated that phase reset was constrained to the theta band. Phase reset was not observed in beta or high-gamma frequencies.

During the sustained response, we resolved the spatiotemporal topography of high-gamma power along the mediolateral extent of HG/TTS (Fig. 3f). A traveling wave of cortical activity coincided with each acoustic pulse, beginning at medial HG/TTS adjacent to the inferior circular sulcus of the insula and propagating laterally across the supratemporal plane to the lip of the lateral fissure (Supplementary Movie 1). Each wave began approximately 80 ms before the acoustic pulse maximum and ended approximately 80 ms afterwards, traversing HG/TTS at a speed of 0.1 m/s. While such spatial organization of neural activity is thought to be important for optimizing cortical computations^22^, prior reports of traveling waves in humans have been confined to lower frequencies^23^ and sleep studies^24^. The wave observed here is considerably slower than would be expected as a simple consequence of the cochlear onset latency gradient^25^.

### Prediction of low-level acoustic stimuli

**Fig. 4:**
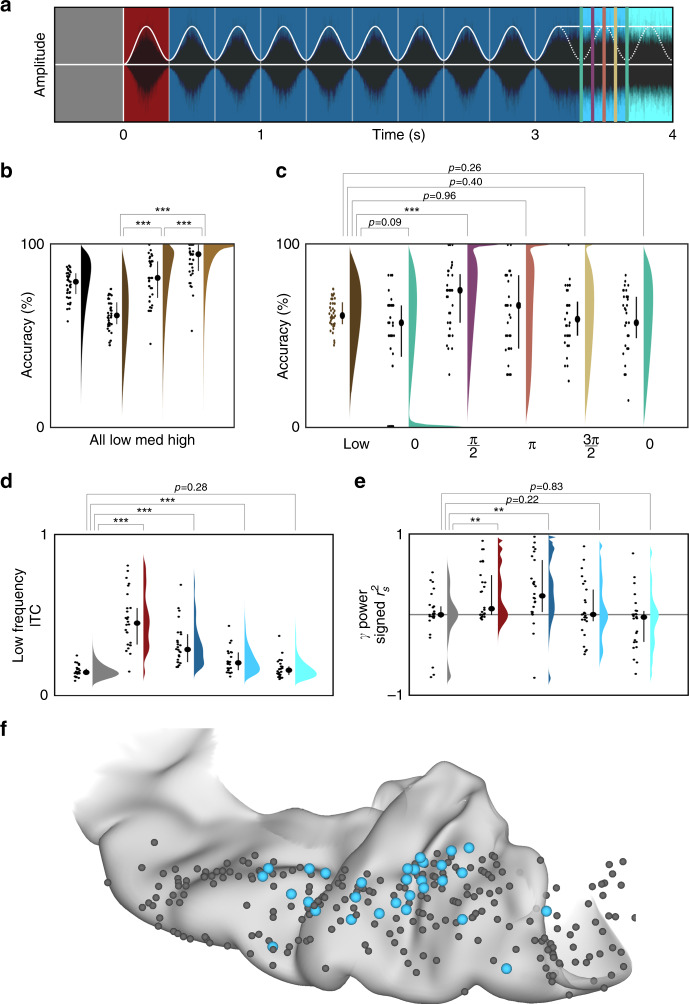
Prediction of timing in auditory cortex. Low-frequency phase in early auditory cortex shows evidence of predictive encoding. **a** The stimulus was divided into five intervals: baseline (gray), onset (red), sustained (dark blue), early prediction (medium blue), and late prediction (light blue). Crucially, there is no modulation of white noise amplitude in either of the prediction intervals. Tones were presented in 50% of trials at one of three intensities and one of five temporal delays. **b** Performance accuracy at each intensity level, grouped across delay categories. For each condition, the raw data (left), interquartile range (middle), and kernel density estimate (right) are shown. Significance was determined in **d**, **e** with the Wilcoxon signed-rank test (*p < 0.05, **p < 0.01, ***p < 0.001). **c** Accuracies in the low-intensity condition separated by temporal delay. Behavioral performance was uniquely increased at the first “missing” acoustic pulse edge—coincident with the phase reset we observe in neural low-frequency response. **d**, **e** The same electrode group shown in Fig. 1b was used for the following analyses. Violin plots demonstrating engagement of low-frequency phase and high-gamma power during each interval. The sustained response in low-frequency phase was measured as average ITC; in high-gamma power, it was measured as a signed *r*^2^ from the Spearman’s correlation with a 3-Hz sine wave. Both low-frequency phase and high-gamma power were significantly engaged during the onset and sustained intervals (*p* < 10^−3^, *p* < 10^−3^; *p* = 0.0012, *p* = 0.0017), but only low-frequency phase remained significantly engaged during the first prediction interval (*p* < 10^−3^). **f** All electrodes on the supratemporal plane (*n* = 349) were evaluated for a significant sustained response during the rhythmic stimulus followed by low-frequency phase reset in the first prediction interval. Those with a significant predictive effect are shown in blue (*n* = 36), predominantly found in HG/TTS.

Patient performance in the one detection task yielded evidence for the extension of the sustained response to subsequent rhythmic prediction (Fig. 4a). Accuracy increased with tone intensity, confirming that patients were engaged and that detectability was limited by the masking noise (Fig. 4b). We then examined the most challenging condition—low-intensity tones—for a variation in detectability modulated by temporal position (Fig. 4c). If the rhythmic noise had no lasting effect on perceptual threshold, the detection rate would be equal across temporal positions. Instead, we found that detection was uniquely improved for the second temporal position, corresponding to the rising slope of the first missing pulse. Notably, this was the same acoustic feature—the rising edge—encoded by low-frequency phase reset in HG/TTS.

To isolate neural mechanisms supporting prediction in HG/TTS, we evaluated the persistence of the sustained response signature to a 3 Hz acoustic envelope after the stimulus rhythm ceased (Fig. 4a). Low-frequency phase maintained the sustained state for one cycle after the last acoustic pulse (Fig. 4d); by the second cycle, the temporal organization of cortical phase was not significantly distinct from pre-stimulus baseline. In contrast, the relationship between high-gamma power and the acoustic envelope did not carry predictive information in either cycle after the last acoustic pulse (Fig. 4e). Thus, prediction in early auditory cortex (Fig. 4f) is best modeled by low-frequency phase reset at acoustic edges. We validated that this predictive effect is not an artifact of filter choice by replicating our findings with a variety of filter implementations (Supplementary Fig. 3). This neural mechanism is engaged within a single cycle of a rhythmic acoustic stimulus and remains active for at least one cycle afterwards. Such a neuro-computational solution for coupled perception and prediction provides a neurobiological basis for cognitive models of speech perception^5–7,20^.

### Cortical response to natural language speech

**Fig. 5:**
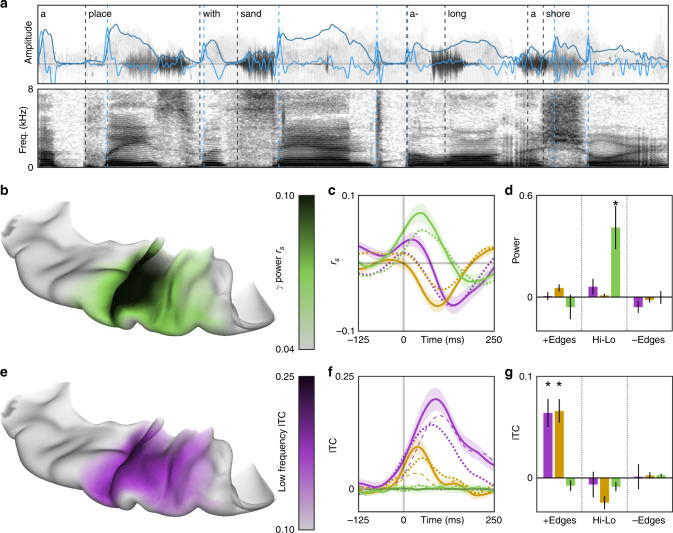
Essential feature encoding of speech in early auditory cortex. Edge detection and envelope tracking during natural language speech occurs focally in early auditory cortex. **a** Patients (*n* = 25) listened to short sentences describing common objects, an example of which is represented here as a time series (top) and a spectrogram (bottom). Two features were extracted from each stimulus: acoustic envelope (light blue) and acoustic edges (dark blue). For comparison, syllabic onsets (black) were also demarcated. **b** The peak lagged Spearman’s correlation between acoustic and high-gamma envelopes and **e** the average low-frequency ITC following an acoustic edge were computed for all electrodes in superior temporal gyrus. These measures were mapped onto a standard MNI atlas, revealing acoustic encodings limited to early auditory cortex. **c** The encoding of acoustic envelope for speech perception was further explored by cross correlation with low frequency (purple), beta (yellow), and high-gamma (green) amplitudes. The effect size was slightly reduced for reversed speech (dotted lines). Shaded area represents ±1 standard error of the mean. **f** Acoustic edges were better encoded by low-frequency phase than syllabic onsets (dashed lines). **d**, **g** In 21 patients with depth electrodes in Heschl’s gyrus, we directly compared the power and phase encodings of discrete acoustic signal events: the difference between 50 ms pre- and post-edge windows, as well as the difference between 50 ms windows centered on envelope peaks and valleys. Significance was evaluated at the group level using the Wilcoxon signed-rank test (*p < 0.001, black bars represent ±1 standard error of the mean). Phase in low frequencies encoded only encoded positive acoustic edges. Power in high-gamma frequencies only encoded the raw envelope highs and lows.

In a second experiment, patients (*n* = 25) named common objects cued by short spoken descriptions (e.g. they heard “a place with sand along a shore” and articulated “beach”)^26^. For each sentence (Fig. 5a), we extracted a pair of key features suggested by our analysis of rhythmic white noise: acoustic envelope and edges. The former describes the instantaneous amplitude of speech, while the latter demarcates moments of rapid amplitude gain. We evaluated the engagement of neural substrates with a sustained response to the white noise stimulus during natural language speech. The cortical encoding of the speech envelope (Fig. 5b) and of edges (Fig. 5e) was localized to HG/TTS—the same supratemporal region that exhibited a sustained response and predictive signature for the white noise stimulus. Power in HG/TTS was significantly correlated with the acoustic envelope of speech (low frequency, *r*_s_ = −0.0620, *p* < 10^−3^; beta, *r*_s_ = −0.0632, *p* < 10^−3^; high gamma, *r*_s_ = 0.0738, *p* < 10^−3^; Fig. 5c) at a frequency-specific delay (low frequency, 135 ms; beta, 95 ms; high gamma, 45 ms; Fig. 5c). Low-frequency phase organization in HG/TTS was significantly increased during the 125 ms following acoustic edges in speech (*p* < 10^−3^; Fig. 5f). Furthermore, it was significantly greater following acoustic edges than following syllabic onsets (*p* = 0.0072; Fig. 5f)—a similar characteristic, but derived from and specific to speech. The correlation between high-gamma activity and the speech envelope was significantly stronger at the electrodes that best tracked the white noise envelope (Fig. 3a) than at neighboring electrodes (*p* < 10^−3^). Similarly, the increase in low-frequency phase organization following acoustic edges in speech was significantly greater at electrodes demonstrating a predictive effect in the white noise task (Fig. 4f) than at their neighbors (*p* < 10^−3^). These findings are concordant with the frequency-multiplexed encoding of acoustic envelope and the low-frequency phase reset at acoustic edges observed during the white noise stimulus. The neural response to acoustic edges was preserved during reversed speech, emphasizing the sublexical nature of this process.

Natural language speech recruited a much broader set of neuroanatomic substrates than white noise, including planum polare, lateral superior temporal gyrus, and superior temporal sulcus (Supplementary Movie 2). In a patient with both surface grid and depth electrodes, only speech induced significant activity in the lateral temporal grid electrodes (Supplementary Fig. 1c). This higher-order auditory cortex is presumably engaged in the processing of higher-order features, both acoustic and language-related (e.g. phonemes^27^).

### Sustained and transient responses in distinct substrates

**Fig. 6:**
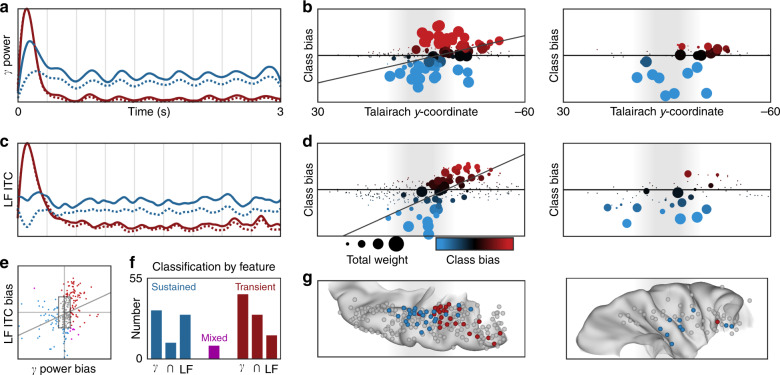
Supratemporal distribution of sustained and transient responses to white noise. Supratemporal responses (*n* = 349 electrodes) classified with two-basis non-negative matrix factorization. **a** High-gamma power identifies a sustained (blue) and transient (red) response: normalized basis functions (dotted line) and the normalized group-average response for the top 10% of electrodes in each class (solid line). **b** Spatial distribution of activation (sum of class weights; point size) and bias (difference in class weights; point color) reveals anteroposterior gradient of functional response. The left panel shows electrodes in language-dominant cortex; the right, in language non-dominant cortex. **c**, **d** Separately, low-frequency ITC also revealed sustained and transient responses with the same spatial distribution. **e** The class bias determined by high-gamma power and low-frequency ITC analyses were significantly correlated. **f** Class biases greater than a value of 10 generated discrete classifications: sustained (*n* = 74), transient (*n* = 90), or mixed (*n* = 9). **g** Electrode classifications are shown on a standard supratemporal atlas, demonstrating a clear functional split between Heschl’s gyrus and planum temporale in language-dominant cortex.

Immediately posterior to HG/TTS in the planum temporale (PT; Fig. 2b; Electrode 2), a distinct functional region generated a transient response to white noise (Fig. 2c). This region featured a high-magnitude increase in high-gamma power accompanied by broadband low-frequency phase reset that returned to pre-stimulus baseline activity after a single acoustic pulse. We separated this transient response the sustained response using non-negative matrix factorization (NNMF)—an unsupervised clustering algorithm uninformed by anatomical position—across all supratemporal electrodes (*n* = 349, Fig. 6a, c). This analysis revealed a distinct anteroposterior response gradient from sustained activity in HG/TTS to transient activity in PT (Fig. 6b, d, g). This spatial distribution was significant for both high-gamma power (Fig. 6b; *r*_s_ = 0.4101, *p* < 10^−4^) and low-frequency phase (Fig. 6d; *r*_s_ = 0.7356, *p* < 10^−16^). Classification by both measures were strongly correlated (Fig. 6e; *r*_s_ = 0.4188, *p* < 10^−6^); only 9 of 349 electrodes showed a mixed classification (i.e. sustained bias in high-gamma power with transient bias in low-frequency phase, or the reverse; Fig. 6f). The sustained response was primarily characterized by *either* high-gamma power (*n* = 33 electrodes) or by low-frequency phase (*n* = 30 electrodes), with only 11 electrodes engaging both measures. This effect was confirmed with analysis of the Kullback–Leibler divergence from a uniform (e.g. non-modulated) activity response (Supplementary Fig. 3). The sustained response was noted in both language-dominant and non-dominant cortex, but the transient response was limited to language-dominant cortex (Fig. 6g).

The spatial topology of early auditory cortical responses was further elucidated within a single patient who underwent two separate implants (Supplementary Fig. 1a): one with surface grid electrodes and another with depth electrodes. Strong sustained encoding in HG/TTS and a robust transient response in PT were observed at electrodes along the supratemporal depth probe (Supplementary Fig. 1b), but not at *any* subdural electrodes directly overlying superior temporal gyrus (Supplementary Fig. 1c). In contrast to prior work using only surface grid electrodes^28^, this unique case indicates that the sustained and transient responses to sublexical features are selectively encoded in early auditory cortex—not in lateral superior temporal gyrus.

### Functional dissociations in speech perception and production

**Fig. 7:**
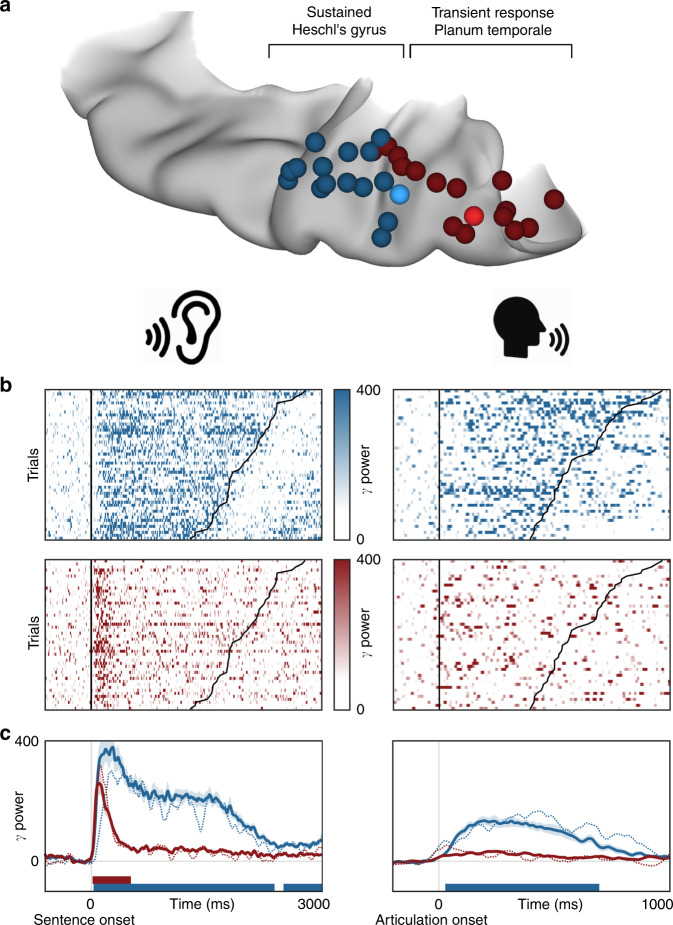
Distinct supratemporal responses during speech listening and articulation. Functional dissociation in HG/TTS and PT during listening and speaking. **a** A pair of electrodes were selected in each patient for a sustained (blue) and transient (red) response to the 3 Hz amplitude-modulated white noise stimulus. One representative patient was highlighted (bright electrode pair) for single-trial analysis. **b** Single-trial raster plots of the percent change in high-gamma power during speech listening and production. **c** High-gamma power averaged across trials and then across patients. Significance was determined with the Wilcoxon signed-rank test at an alpha level of *p* < 0.001 using familywise error correction. The mean activity from the representative individual highlighted in panel **b** is included as the dotted line. While the high-gamma response in HG/TTS is reduced during articulation, the response in PT is eliminated.

We compared neural activity in both HG/TTS and PT during listening and speaking—externally and internally generated speech. In each patient with a supratemporal depth probe, the pair of electrodes with the strongest sustained and transient responses were identified during the rhythmic white noise condition. These criteria selected electrodes in HG/TTS and PT, respectively (Fig. 7a). High-gamma power in these regions was analyzed relative to sentence and articulation onset for a representative individual (Fig. 7b) and across the group (Fig. 7c). HG/TTS responded strongly during both listening and speaking, remaining active for the duration of each sentence and throughout articulation. In contrast, PT also responded strongly following sentence onset with peak activity at 100 ms; however, this region was quiescent during articulation (Supplementary Movie 3).

**Fig. 8:**
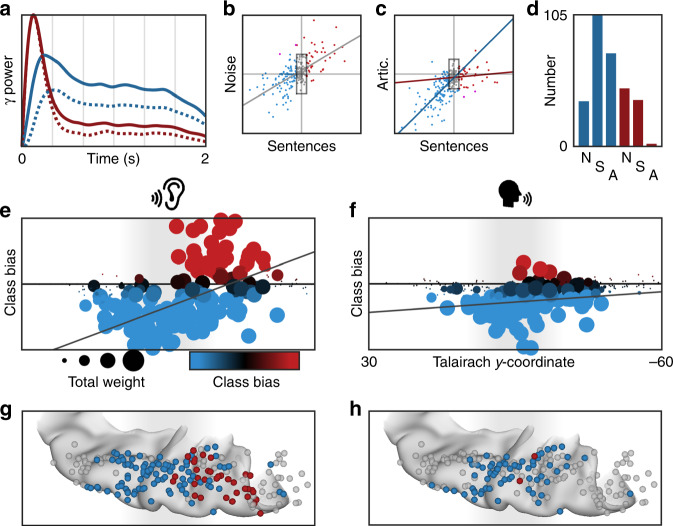
Planum temporale responses are uniquely suppressed for self-generated speech. Supratemporal responses (*n* = 247 electrodes) classified with 2-basis non-negative matrix factorization. **a** High-gamma power identifies a sustained (blue) and transient (red) response: normalized basis functions (dotted line) and the normalized group-average response for the top 10% of electrodes in each class (solid line). **b** The class bias determined by factorizations of electrode responses to noise and sentence listening—homogeneous and structure acoustic inputs—were significantly correlated. **c** The factorization from sentence listening was applied to the electrode responses at articulation. Sustained class bias was significantly correlated for listening and speaking, but the transient class biases were uncorrelated. **d** Class biases greater than a value of 10 generated discrete classifications for speech listening (S; sustained, *n* = 105; transient, *n* = 37) and articulation (A; sustained, *n* = 74; transient, *n* = 2). **e**, **f** Spatial distribution of activation (sum of class weights; point size) and bias (difference in class weights; point color) reveals anteroposterior gradient of functional response during speech listening (left) but not articulation (right). **g**, **h** Electrodes shown on a standard supratemporal atlas reveal that the transient response is localized to PT and is uniquely suppressed during articulation.

We further characterized the spatial distribution of the transient response during speech listening and its suppression during speech production using NNMF. As for the white noise stimulus, high-gamma power yielded sustained and transient response types (Fig. 8a) along a robust anteroposterior distribution (Fig. 8e, g; *r*_s_ = 0.4688, *p* < 10^−10^). These were strongly correlated with the class biases for white noise listening (Fig. 8b; *r*_s_ = 0.5849, *p* < 10^−23^). When this factorization was applied to high-gamma power during articulation (Fig. 8c, f, h), the sustained response was preserved (*r*_s_ = 0.6663, *p* < 10^−16^) while the transient response type was suppressed (*r*_s_ = 0.1094, *p* = 0.3279). Of the 37 electrodes demonstrating a transient response during speech listening, only 2 retained this classification during articulation (Fig. 8d). The functional dissociation at PT between externally and internally generated speech is consistent with the theory of predictive coding during speech production^20^ via motor-to-sensory feedback^15,16,29^.

### Stimulation mapping of the supratemporal plane

In three patients, language mapping using direct cortical stimulation was performed along the full extent of the supratemporal plane. Current was passed between adjacent pairs of electrodes, transiently mimicking a focal lesion. Three language tasks were used to identify eloquent cortex: spoken sentence repetition, spoken naming to definition, and picture naming.

Direct cortical stimulation revealed distinct functional deficits in HG/TTS and PT (Supplementary Table 1). Stimulation of HG/TTS disrupted speech comprehension impacting both sentence repetition and naming to definition; however, picture naming was unaffected. Patients reported stimulation-evoked auditory phenomena including “buzzing” and “ringing.” Stimulation of PT disrupted articulation in all tasks, including picture naming. Furthermore, it evoked auditory hallucinations that included the sensation that “somebody’s talking” or “people [are] talking all around me … like a ballpark”, the abstract ideation of “a rolling of more words”, and an “echo” like the speaker was “underwater.” These hallucinations were not induced by stimulation of the lateral superior temporal gyrus.

**Fig. 9:**
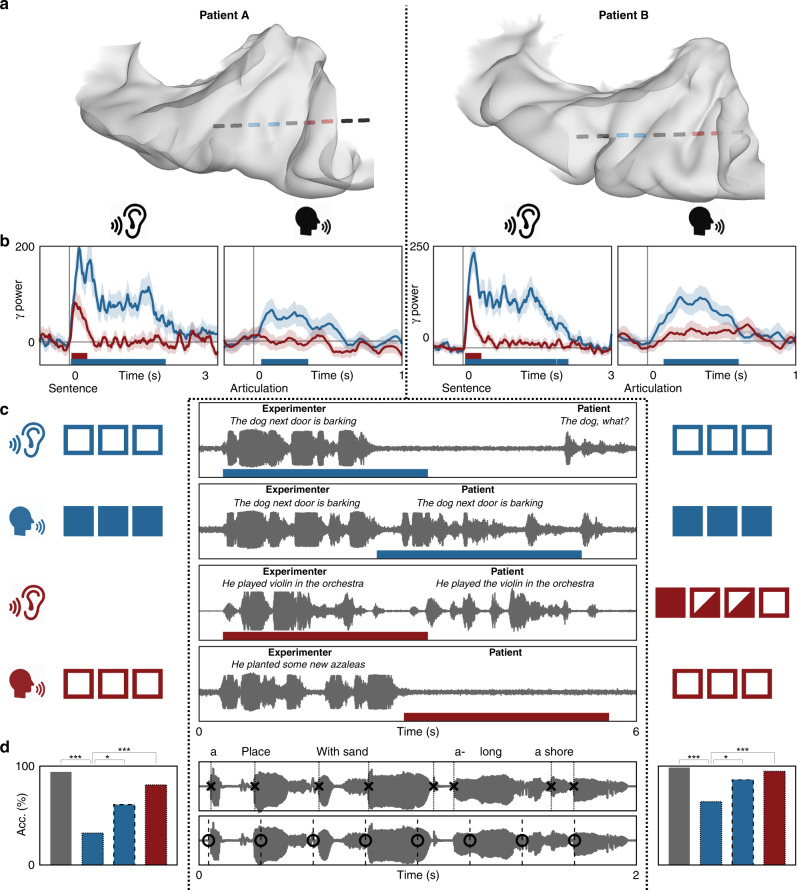
Chronometric stimulation of the supratemporal plane. Supratemporal responses during two chronometric stimulation experiments. The first patient is shown on the left and the second on the right. **a** Electrode positions relative to patient-specific neuroanatomy. Two pairs of electrodes localized to HG/TTS and PT demonstrated sustained (blue) and transient responses (red), respectively. **b** Average percent change in high-gamma power during auditory naming to definition: sentence listening (left) and articulation (right). The transient response is entirely suppressed during articulation. **c** Chronometric cortical stimulation mapping with 50 Hz balanced 0.3 ms period square waves for 3 s during sentence repetition. Successful trials are indicated by full boxes, failed trials by empty boxes, and trials with a single error (e.g. word replacement) by half-full boxes. Stimulation was delivered either at stimulus onset (top panel) or offset (middle and bottom panel). The patient was unable to repeat the stimulus when HG/TTS was stimulated during listening, but was unaffected when the HG/TTS was stimulated during production. In contrast, stimulation of PT during production induced failure. **d** Chronometric stimulation during auditory naming to definition at either acoustic edges (x’s; dotted lines) or uniformly distributed times (o’s; dashed lines). Baseline accuracies were 94 and 99% (gray bar). Stimulation at acoustic edges in HG/TTS (left blue bar) and PT (red bar) resulted in naming accuracies of 32 and 81% in the first patient and 64 and 95% in the second patient. Stimulation of HG/TTS delivered uniformly throughout the stimulus (right blue bar) resulted in naming accuracies of 61 and 86%. Significant differences between binomial distributions were evaluated via *z* statistic (*p < 0.05, **p < 0.01, ***p < 0.001).

Prospectively, we implemented two stimulation experiments in two additional patients. The rhythmic white noise and auditory naming experiments were used to identify electrodes with sustained and transient responses in HG/TTS and PT, respectively (Fig. 9a, b). In the first stimulation experiment (Fig. 9c), the patients were asked to repeat a spoken sentence. The same stimulation current, frequency, and waveform used in the clinical standard above was applied at either the onset or the offset of the stimulus, disrupting processes related to comprehension or articulation, respectively. We found that stimulation of HG/TTS at stimulus onset interrupted speech perception, while the same stimulation at stimulus offset had no effect on articulation. In contradistinction, stimulation of PT at stimulus offset resulted in articulatory failure (Supplementary Movie 4). These findings causally confirm the separable roles of HG/TTS and PT revealed by our analyses of passive electrocorticographic recordings.

In the second experiment (Fig. 9d), patients performed naming to definition with concurrent chronometric stimulation. During the presentation of each spoken description, stimulation was triggered at either acoustic edges or at a uniform rate. Stimulation intensity was greatly reduced from clinical mapping parameters; only a single 500-µs wide square wave pulse was delivered with each trigger (average 6.9 triggers per trial). Stimulation of HG/TTS at acoustic edges resulted in significantly worse task performance than equivalent stimulation of PT (32% vs. 81%, *p* < 10^−3^; 64% vs. 95%, *p* < 10^−3^). Furthermore, stimulation of HG/TTS at acoustic edges also resulted in significantly worse task performance than uniformly distributed stimulation matched for total delivered current (32% vs. 61%, *p* = 0.0246, 64% vs. 86%, *p* = 0.0397). Patients exhibited baseline accuracies of 94 and 99% without stimulation during the task. These results further causally corroborate our findings that HG/TTS is especially attuned to acoustic edges and that PT is not engaged for speech comprehension.

## Discussion

These large-scale direct intracranial recordings and stimulations of the supratemporal plane have revealed the functional architecture of prediction in human early auditory cortex at fine-grained resolution. We demonstrate that a sustained response to speech engages a frequency-multiplexed encoding of two sublexical features: envelope and edges. We also uncover a pair of distinct neuroanatomic substrates that perform two separate types of prediction: temporal prediction in bilateral HG/TTS and speech-specific suppression in language-dominant PT. The identification and characterization of these mechanisms advances the understanding of how human cerebral cortex parses continuous acoustic input during both speech perception and production.

Using electrodes positioned along the anteroposterior extent of the supratemporal plane, we localized the cortical signature of a sustained response during listening to strictly early auditory cortex: Heschl’s gyrus and the transverse temporal sulcus^30^. This signature was considerably more complex than that suggested by prior studies, comprising a frequency-multiplexed encoding of envelope phase—distinct for rising and falling amplitudes of the same magnitude—in low frequency, beta, and high-gamma power. We also identified a separate, concurrent encoding of acoustic edges in low-frequency phase reset. This latter encoding uniquely persisted after the rhythmic stimulus ended, consistent with the behavior of a predictive neural mechanism (and inconsistent with the behavior of evoked response potentials).

Importantly, identical cortical substrates engaged the same mechanisms during natural sentence listening. The speech envelope was tracked by bandlimited power and acoustic edges were demarcated by low-frequency phase reset. Furthermore, acoustic edges were more strongly encoded in the cortical phase than syllabic onsets—a linguistic feature with similar frequency and periodicity. This supports the assertion^31^ that these mechanisms are driven by sublexical acoustic processing, perhaps even inherited from subcortical regions (e.g. medial geniculate nucleus).

The organization of transient excitability states within neuronal populations has been thought of as a gating mechanism for cortical columns^32–34^. Discrete high excitability periods constitute windows of opportunity for input into sensory cortex, as evidenced by peri-threshold detection studies in somatosensory^35^, visual^36^, and auditory^31,37,38^ regions. During listening, such windows might serve to segment speech to facilitate comprehension^39^. More generally, the temporal organization of high excitability periods could serve to minimize temporal uncertainty in stimulus processing and detection^4,40^. This view was corroborated by a behavioral study of responses to the same white noise stimulus used in these experiments that revealed a striking relationship between detection accuracy and the preceding rhythmic stimulus^41^. With the direct intracranial recordings in this study, we found that low-frequency phase reset anticipates the first missing acoustic edge. These results constitute strong evidence for neural mechanisms in early auditory cortex supporting temporal prediction, a fundamental computational element in models of speech perception^5–7,20^. Our results are consistent with the predictive encoding of when by a bandlimited complex of discrete computational channels, each arising from distinct patterns of hierarchical cortical connectivity^20^.

Entrainment—the synchronization of intrinsic neural oscillations with extrinsic rhythmic signals—has been suggested to have an important role in a variety of cognitive processes including attentional selection^4,8,42^ and internal timekeeping^36,43^. Entrainment has also been implicated in speech perception by evidence that envelope distortions impair comprehension^44,45^ independent of spectral content^46,47^ and that the degree of neuro-acoustic entrainment modulates intelligibility^48,49^. Simultaneously, the very existence of entrainment remains the subject of ongoing debate—specifically, whether entrainment can be more simply explained as a recurring series of transient evoked responses^50,51^. We find the sustained response to be comprised of a multispectral non-adapting state that is distinct from the evoked response at stimulus onset and that endures in part after the stimulus ends. While concurrent thalamocortical recordings are probably necessary to definitively separate entrainment from evoked response potentials, we demonstrate the neural mechanisms active during the sustained state are foundational to the hierarchy of acoustic perception—including language.

While the sustained response was constrained to Heschl’s gyrus and the transverse temporal sulcus, we observed a distinct transient response in planum temporale. The transient response was characterized by a brief spike in high-gamma power and rapid reset of low-frequency phase immediately following acoustic onset. Interestingly, this response was not engaged during self-generated speech. Such preferential engagement for unexpected sound is consistent with predictive encoding during speech production^15,16^. Upon execution of a speech motor plan, a learned internal model generates an efference copy^52,53^—an expected sensory result. When the acoustic input matches this efference copy, no cortical signal is generated; however, when a mismatch occurs (e.g. externally generated sound or speech), an error signal results^20^. This is precisely what we observed in the planum temporale, distinct from the sustained response in Heschl’s gyrus. We further corroborated these results with direct current injection at Heschl’s gyrus and planum temporale; stimulation of the former area selectively disrupted speech perception, while stimulation of the latter area selectively disrupted speech production.

There is no direct evidence for internal predictive models instantiated in human cortex^54^. Our results advance understanding of the neurobiology of predictive speech coding in two respects. First, functional studies have revealed single-unit preference in primary auditory cortex for listening or speaking in both non-humans^55^ and humans^52^. It has recently been asserted that these response tunings overlap—an “intertwined mosaic of neuronal populations”^56^ in auditory cortex. Instead, the complete anteroposterior mapping of the supratemporal plane in a large patient cohort enabled us to identify a distinct neuroanatomical organization in planum temporale. Second, several groups report cortical response suppression specific for self-generated speech^56–58^. We reveal two distinct modes that enable this suppression: a partial reduction of activity in Heschl’s gyrus and a complete absence of the transient response in planum temporale. The stapedius reflex^55^ does not explain the latter mode, suggesting a neural mechanism of suppression. All together, we provide compelling evidence for efference copies—predictive encoding of what^20^—and their essential role in speech production^15,16^.

## Methods

### Population

Thirty-seven patients (20 males, 16 females; mean age 33 ± 9; mean IQ 97 ± 15) undergoing evaluation of intractable epilepsy with intracranial electrodes were enrolled in the study after obtaining informed consent. Study design was approved by the committee for the protection of human subjects at the University of Texas Health Science Center. A total of 7507 electrodes (6669 depths, 838 grids) were implanted in this cohort. Only electrodes unaffected by epileptic activity, artifacts, or electrical noise were used in subsequent analyses.

Hemispheric language dominance was evaluated in all patients with intra-carotid sodium amytal injection^59^ (*n* = 5), functional magnetic resonance imaging (fMRI) laterality index^60^ (*n* = 7), cortical stimulation mapping^61^ (*n* = 12), or the Edinburgh Handedness Inventory^62^ (*n* = 13). Thirty-two patients were confirmed to be left-hemisphere language-dominant. Two patients were found to be left-handed by EHI and did not undergo alternative evaluation; they are assumed to be left-hemisphere dominant, but were excluded from laterality analysis. Three patients were found to be right-hemisphere language-dominant; two by intra-carotid sodium amytal injection, and one by fMRI laterality index. For representational purposes, language-dominant supratemporal electrodes in these patients were mirrored onto the same left supratemporal cortical model as left-hemisphere language-dominant patients.

### Paradigms

Two distinct paradigms were used. The first experiment featured amplitude-modulated white noise, while the latter experiment contained natural speech. All were designed to evaluate the response of early auditory cortex to external acoustic stimuli. Stimuli were played to patients using stereo speakers (44.1 kHz, 15″ MacBook Pro 2013) driven by either MATLAB R2014 (first experiment) or Python v2.7 (second experiment) presentation software.

The first experiment presented patients with a single-interval two-alternative forced-choice perceptual discrimination task. The stimulus comprised two periods. In the first, wideband Gaussian noise was modulated (3 Hz, 80% depth) for 3 s. In the second, the modulation waveform ended on the cosine phase of the next cycle to yield 833 ms of constant-amplitude noise. Furthermore, 50% of trials featured a peri-threshold tone (1 kHz, 50 ms duration, 5 ms rise-decay time) that was presented at one of five temporal positions and at an amplitude level from one of three values. The temporal positions were separated by a quarter cycle of the modulation frequency beginning with the constant-amplitude noise. The amplitude levels covered a range of 12 dB. On each trial, the patient was required to indicate via a key press whether a tonal signal was present during the unmodulated segment of the masking noise. All 37 patients each completed 100 trials. Seven patients also underwent testing with stimulus modulation frequencies of 5 and 7 Hz.

In the second experiment, patients engaged in an auditory-cued naming task: naming to definition. The stimuli were single sentence descriptions (average duration 1.97 ± 0.36 s, 7.7 syllables, 6.9 acoustic edges) recorded by both male and female speakers. These were designed such that the last word always contained crucial semantic information without which a specific response could not be generated (e.g., “A round red *fruit*”)^63^. Patients were instructed to articulate aloud the object described by the stimulus. All patients achieved greater than 90% accuracy (average accuracy 93%, average reaction time 1.08 s). In addition, temporally reversed speech was used as a control condition. These stimuli preserved the spectral content of natural speech, but communicated no meaningful linguistic content. For each stimulus, patients were instructed to articulate aloud the gender of the speaker. Twenty-five patients each completed 180 trials.

### MR acquisition

Pre-operative anatomical MRI scans were obtained using a 3 T whole-body MR scanner (Philips Medical Systems) fitted with a 16-channel SENSE head coil. Images were collected using a magnetization-prepared 180° radiofrequency pulse and rapid gradient-echo sequence with 1 mm sagittal slices and an in-plane resolution of 0.938 × 0.938 mm. Pial surface reconstructions were computed with FreeSurfer (v5.1)^64^ and imported to AFNI^65^. Post-operative CT scans were registered to the pre-operative MRI scans to localize electrodes relative to cortical landmarks. Grid electrode locations were determined by a recursive grid partitioning technique and then optimized using intra-operative photographs^66^. Depth electrode locations were informed by implantation trajectories from the ROSA surgical system.

### ECoG acquisition

Stereo-electroencephalographic depth probes with platinum-iridium electrode contacts (PMT Corporation; 0.8 mm diameter, 2.0 mm length cylinders; adjacent contacts separated by 1.5–2.43 mm) were implanted using the Robotic Surgical Assistant (ROSA; Zimmer-Biomet, Warsaw, IN) registered to the patient using both a computed tomographic angiogram and an anatomical MRI^67^. Each depth probe had 8–16 contacts and each patient had multiple (12–16) such probes implanted. Surface grids—subdural platinum–iridium electrodes embedded in a silastic sheet (PMT Corporation, Chanhassen, MN; top-hat design; 3 mm diameter cortical contact)—were surgically implanted via a craniotomy^68^. ECoG recordings were performed at least 2 days after the craniotomy to allow for recovery from the anesthesia and narcotic medications. Thirty-five patients were implanted with depth probe electrodes; four patients were implanted with surface grid electrodes. Notably, a pair of patients had two separate implants: first with depth probe electrodes and subsequently with surface grid electrodes. Neural data from depth and surface grid electrodes are comparable given the identical contact material, similar surface area, and identical recording hardware. The relative proportion of depth probe and grid electrode implants reflects the major improvements in patient safety and outcome afforded by recent advances in stereotactic electroencephalography^69^.

Data were collected at a 2000 Hz sampling rate and 0.1–700 Hz bandwidth using NeuroPort NSP (Blackrock Microsystems, Salt Lake City, UT). Stimulus presentation software triggered a digital pulse at trial onset that was registered to ECoG via digital-to-analog conversion (MATLAB: USB-1208FS, Measurement Computing, Norton, MA; Python: U3-LV, LabJack, Lakewood, CO). Continuous audio registered to ECoG was recorded with an omnidirectional microphone (30–20,000 Hz response, 73 dB SNR, Audio Technica U841A) placed adjacent to the presentation laptop. For the naming to definition and reversed speech experiments, articulation onset and offset were determined by offline analysis of the amplitude increase and spectrographic signature associated with each verbal response.

Cortical areas with potentially abnormal physiology were excluded by removing channels that demonstrated inter-ictal activity or that recorded in proximity to the localized seizure onset sites. Additional channels contaminated by >10 dB of line noise or regular saturation were also excluded from further analysis. The remaining channels were referenced to a common average comprised of all electrodes surviving these criteria. Any trials manifesting epileptiform activity were removed. Furthermore, trials for the naming to definition and reversed speech experiments in which the patient answered incorrectly or after more than 2 s were eliminated.

### Digital signal processing

Data were processed with MATLAB R2018b. Line noise was removed with zero-phase second-order Butterworth bandstop filters at 60 Hz and its first two harmonics. The analytic signal was generated with frequency domain bandpass Hilbert filters featuring paired sigmoid flanks (half-width 1 Hz)^70^. For spectral decompositions, this was generalized to a filter bank with logarithmically spaced center frequency (2–16 Hz, 50 steps) and passband widths (1–4 Hz, 50 steps). Instantaneous amplitude and phase were subsequently extracted from the analytic signal. In this fashion, we used both narrowband and wideband analyses to precisely quantify the frequency driving a local cortical response and its timing, respectively.

### Direct cortical stimulation and analysis

The supratemporal plane was clinically evaluated with stimulation mapping in three patients. Concurrent electrocorticographic monitoring was carried out in all cases to detect any induced seizures. Trains of 50 Hz balanced 0.3 ms period square waves were delivered to adjacent electrodes for 3 s during the task^61^. Stimulation was applied using a Grass S88X Stimulator with a SIU (Grass Technologies, West Warwick, RI). At each electrode pair, stimulation was begun at a current of 2 mA and increased stepwise by 1 mA until either an overt phenomenon was observed, after-discharges were induced, or the 10 mA limit was reached. Stimulation sites were defined as positive for language tasks if stimulation resulted in articulation arrest or anomia. Furthermore, stimulation sites causing movement or sensation were separately recorded. Patient responses and behavior were evaluated by clinical experts present in the room during the entire mapping session.

One patient underwent a pair of chronometric stimulation experiments, each performed separately at HG/TTS and PT. In the first experiment, the same clinical stimulation protocol was applied during sentence repetition at either the sentence onset or its conclusion. In the second experiment, patients attempted the auditory-cued naming task described above while single balanced 0.3 ms period pulses were triggered throughout the spoken description. These triggers were either cued by acoustic edges (HG/TTS, 31 trials; PT, 21 trials) or, as a control, uniformly distributed (18 trials). The latter condition was matched for total current delivered during the entirety of each sentence. Patient performance was quantitatively assessed in both experiments by either repetition or naming success, respectively.

### Statistical analysis

Analyses were performed with trials time-locked to stimulus onset (all experiments) and to articulation onset (only naming to definition and reversed speech experiments). The baseline period for all experiments was defined as −300 to −50 ms relative to stimulus onset. All time traces were smoothed after statistical analysis with a Savitsky–Golay polynomial filter (third order, 83 ms frame length) for visual presentation.

Instantaneous amplitude was squared and then normalized to the baseline period, yielding percent change in power from baseline. Statistical significance against baseline was evaluated with the Wilcoxon signed-rank test. Significance of the power response response at discrete stimulus phase was evaluated using a bootstrapped distribution of Kullback–Leibler divergences from a uniform distribution across temporally jittered trial sequences.

The alignment of instantaneous phase at each trial *θ*_*n*_ was quantified with inter-trial coherence (ITC) **L**, defined as follows where *N* is the number of trials:

1 $${\mathbf{L}} = \frac{1}{N}\mathop {\sum}\limits_{n = 1}^N {{\mathrm{{e}}}^{i\theta _n}}.$$

At the group level, significance was evaluated with the Wilcoxon signed-rank test. At the individual electrode level, significance was evaluated using a bootstrapped distribution comprised of ITC across temporally jittered trial sequences.

To visualize the distinct activity states traversed by the auditory cortex, we plotted phase space trajectories for high-gamma power and low-frequency ITC. These trajectories are defined by the group estimate of each measure plotted against itself at a quarter period delay (83 ms). This analysis visualizes the temporal behavior of a dynamical system. Of note, stable systems generate limit cycles—stationary repeating patterns.

NNMF is an unsupervised clustering algorithm^71^. This method expresses non-negative matrix **A** ∈ *R*^mxn^ as the product of class weight matrix **W** ∈ *R*^mxk^ and class archetype matrix **H** ∈ *R*^kxn^, minimizing:

2 $${\mathbf{A}} - {\mathbf{WH}}_F^2.$$

The factorization rank *k* = 2 was chosen for all analyses in this work. Repeat analyses with higher ranks did not identify additional response types. We optimized the matrix factorization with 1000 replicates of a multiplicative update algorithm (MATLAB R2018b Statistics and Machine Learning Toolbox). Two types of inputs were separately factorized: mean high-frequency power and low-frequency phase ITC. High-gamma power and low-frequency phase ITC less than baseline were rectified. These features were calculated for the *m* electrodes in the supratemporal plane at *n* time points. Factorization thus generated a pair of class weights for each electrode and a pair of class archetypes—the basis function for each class. Class bias was defined as the difference between the class weights at each electrode. Response magnitude was defined as the sum of class weight magnitudes at each electrode. Separate factorizations were estimated for white noise listening and for natural speech listening. The latter was then applied to self-generated speech by conserving the class archetypes and recalculating the class weights:

3 $${\mathbf{W}} = {\mathbf{A}}\,{\mathbf{H}}^{ - 1}.$$

Response classifications were established by applying a binary threshold to the class biases. Spearman correlations of class bias and anatomical position were calculated using electrodes with a large response magnitude (>10).

Surface-based mixed-effects multilevel analysis (SB-MEMA) was used to provide statistically robust and topologically precise effect estimates of bandlimited power change from the baseline period^72–74^. This method^75^ accounts for sparse sampling, outlier inferences, as well as intra- and inter-subject variability to produce population maps of cortical activity. SB-MEMA was run on short, overlapping time windows (150 ms width, 10 ms spacing) to generate the frames of a movie portraying cortical activity. All maps were smoothed with a geodesic Gaussian smoothing filter (3 mm full-width at half-maximum) for visual presentation.

### Reporting summary

Further information on research design is available in the Nature Research Reporting Summary linked to this article.

## Supplementary information

Supplementary Information

Reporting Summary

Description of Additional Supplementary Files

Supplementary Movie 1

Supplementary Movie 2

Supplementary Movie 3

Supplementary Movie 4

## Data Availability

The datasets collected and analyzed during the current study are not publicly available as the auditory component is essential for interpretation and constitutes protected information. Grouped data representations are available from the corresponding author on reasonable request. A reporting summary for this Article is available as a Supplementary Figure file. Source data are provided with this paper.

## Source data

Source Data
