# Supplementary material for: Language prediction mechanisms in human auditory cortex
Source: Nat Commun. 2020 Oct 16;11:5240. doi: 10.1038/s41467-020-19010-6 (PMC7567874; doi:10.1038/s41467-020-19010-6)
Supplement: Supplementary file 1 — Supplementary Information [file 41467_2020_19010_MOESM1_ESM.pdf]

Evidence of Language Prediction Mechanisms in Human Auditory Cortex

Forseth et al.

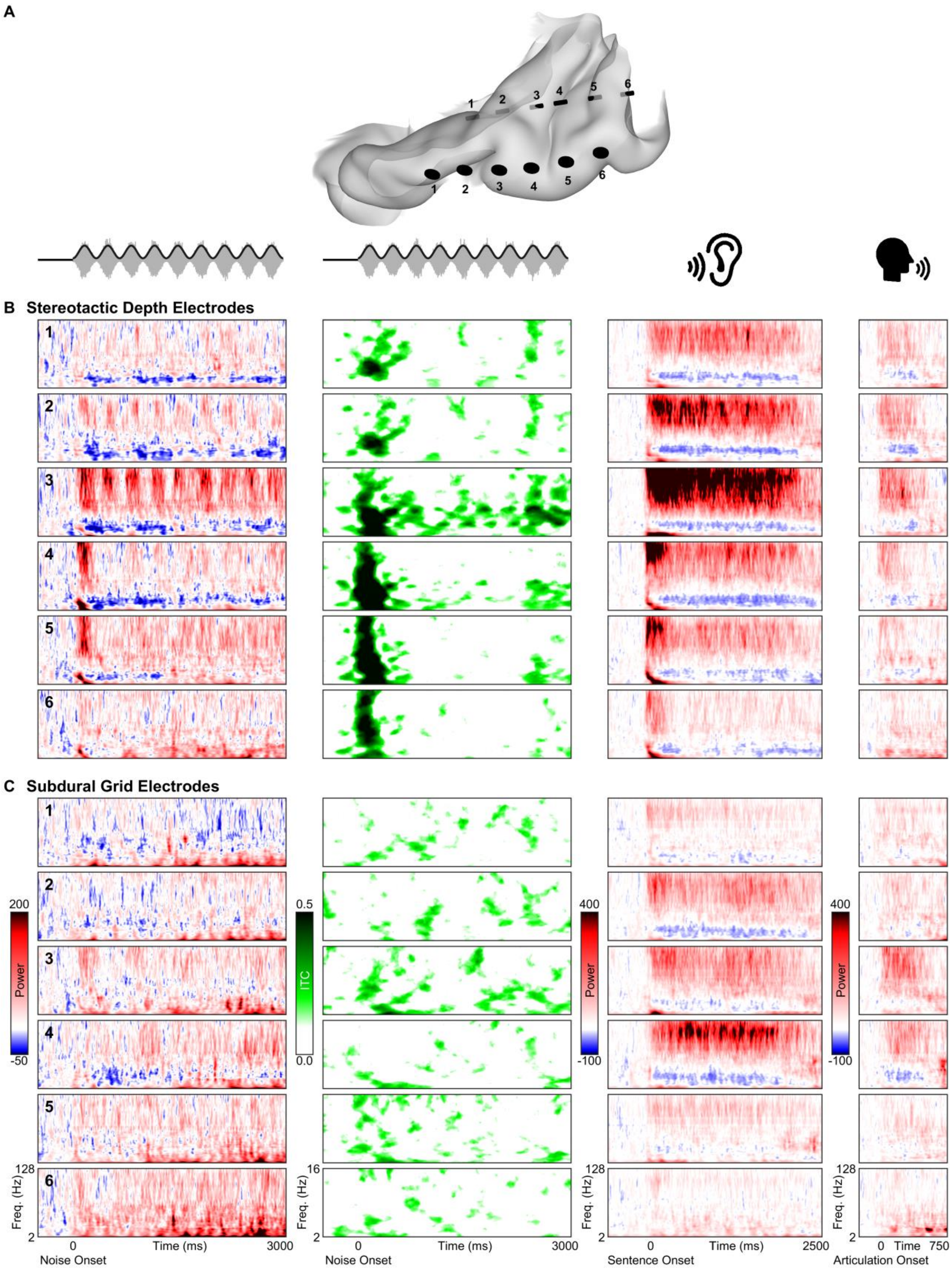

Supplementary Figure 1: Individual Response at Depth and Surface Electrodes to Noise and Speech
Single patient with two separate implants, one with depths & one with grids. (**A**) Electrodes matched by closest distance (<15mm), spanning planum polare through Heschl's gyrus to planum temporale. (**B, C**) Rows arranged from anterior (top) to posterior (bottom). First column, power during 3 Hz stimulus; second column, ITC during 3 Hz stimulus; third column, power during sentence listening; fourth column, power during articulation. The sustained response to noise in HG & the transient response in PT were only observed in depth electrodes – not at surface grid electrodes. Surface grid electrodes only responded to speech.

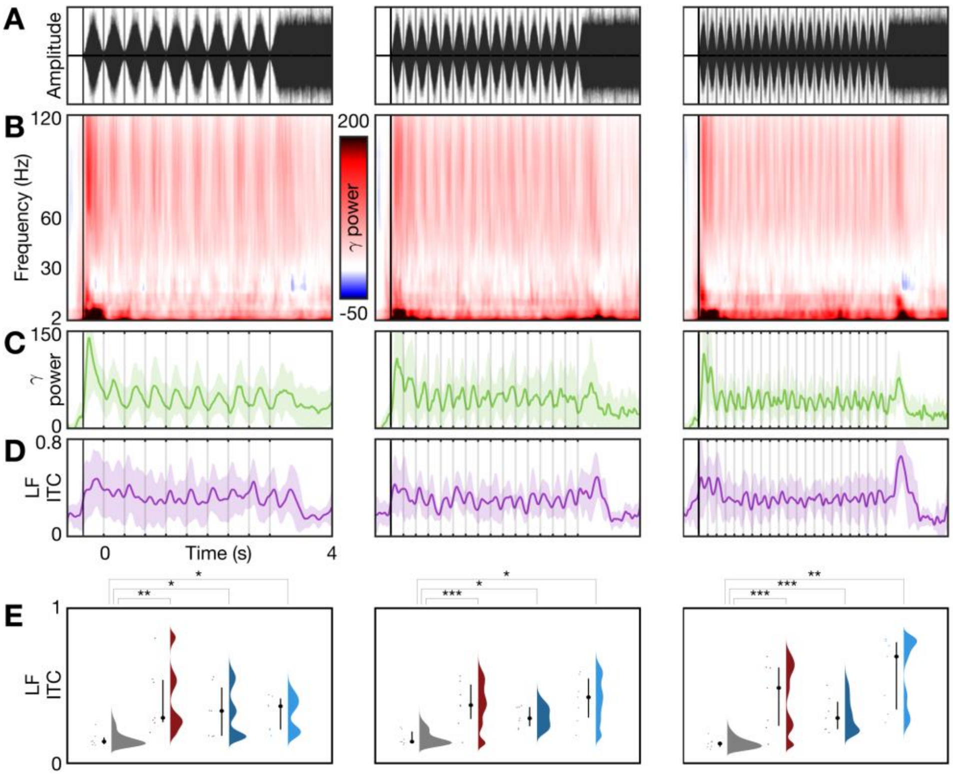

Supplementary Figure 2: Sustained Oscillatory Response at 3, 5, and 7 Hz

The sustained oscillatory response is not specific to a 3 Hz envelope. In 8 patients, we evaluated the sustained response to amplitude-modulated white noise at a range of frequencies: 3 Hz (left), 5 Hz (center), and 7 Hz (right). (**A**) The stimulus waveforms. (**B**) Group-average spectrograms, normalized to baseline, of electrodes in HG/TTS. (**C**) The average high-gamma power response. (**D**) The average low-frequency ITC. Shaded area represents +/- 1 standard error of the mean. (**E**) Violin plots demonstrating the activity in low-frequency phase. Significance was evaluated at the group level using the Wilcoxon signed-rank test (* $p < 0.05$, ** $p < 0.01$, *** $p < 0.001$). There were significant increases in ITC relative to baseline in the initial, sustained, and post-oscillatory periods for 3 Hz ($p = 0.0078$, $p = 0.0391$, $p = 0.0156$), 5 Hz ($p < 10^{-3}$, $p = 0.0156$, $p = 0.0156$), and 7 Hz ($p < 10^{-3}$, $p < 10^{-3}$, $p = 0.0078$) stimuli.

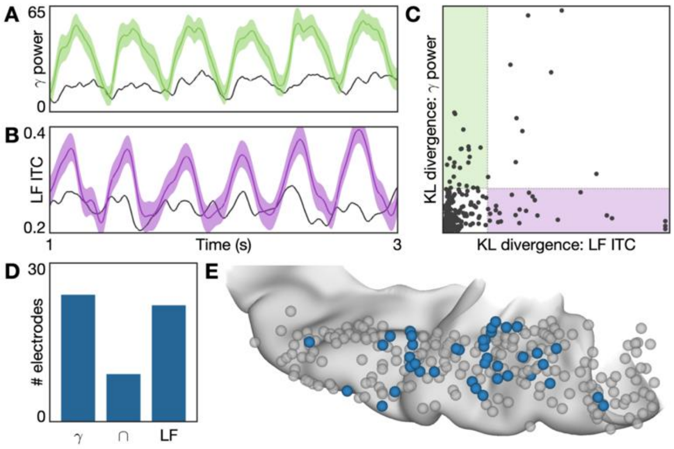

Supplementary Figure 3: Kullback-Leibler Divergence Shows Separate Encodings for Power and Phase

The sustained response was primarily characterized by either rhythmic encoding of high-gamma power or low-frequency phase. We quantified the degree of rhythmicity in power and phase at each electrode as KL divergence from a uniform response. The mean population response of electrodes with a KL divergence of power (**A**) or phase (**B**) greater than 0.1 are shown in color (shaded area represents +/- 1 standard error of the mean); the response of the opposite class (i.e. power response of electrodes with a high KL divergence of phase) is shown in black. (**C**) Scatterplot of power and phase divergence with thresholds indicated in colors matching panels A,B. (**D**) Most active electrodes were either high-gamma selective (n = 24) or low-frequency phase selective (n = 22); only 9 electrodes showed joint rhythmicity in power and phase. (**E**) Electrodes shown on a standard supratemporal atlas revealing a predominant distribution around Heschl’s gyrus.

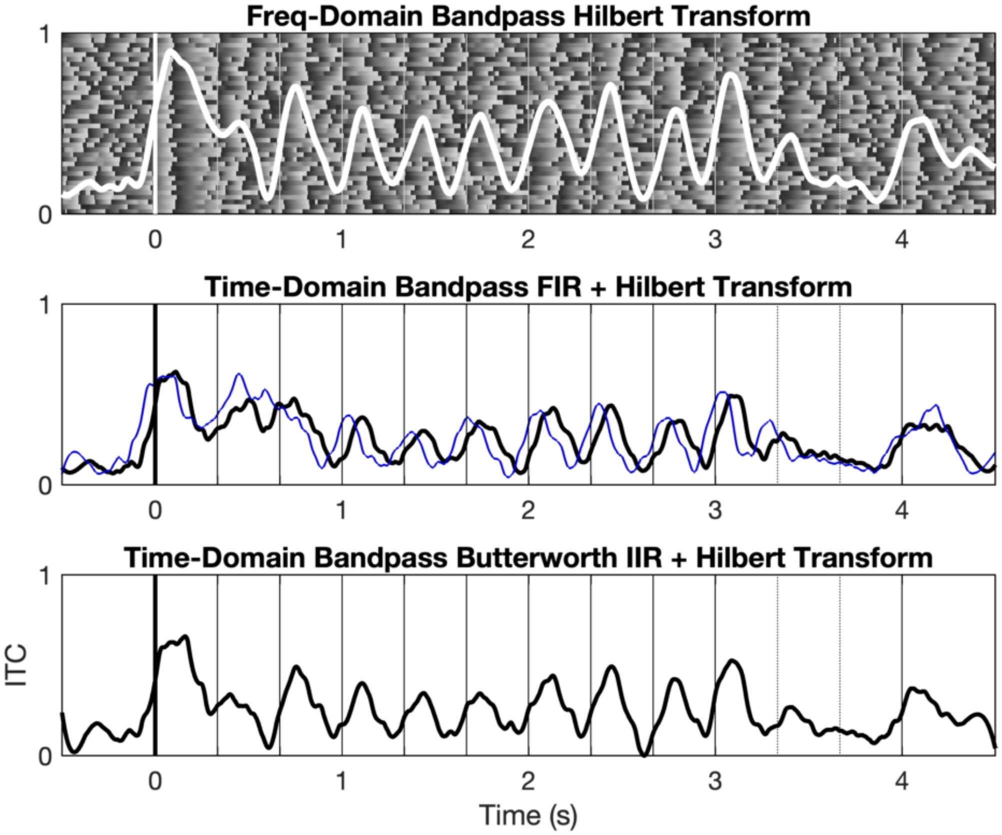

Supplementary Figure 4: Filter Comparison

Filter comparison at a single electrode in Heschl's gyrus demonstrating that the predictive effect is robust to filter design choice. Top row: Frequency domain bandpass Hilbert transform (used throughout manuscript). Background shows instantaneous phase for each trial in rows. Foreground traces shows inter-trial coherence (ITC) – the phase length at each time point across trials. Critically, the predictive effect can be seen at 3.33 – 3.66 seconds; an ITC increase despite the absence of any amplitude modulation. Middle row: Time domain FIR bandpass filter and Hilbert transform with both zero-phase acausal implementation (black trace) and right-filtered causal implementations (blue trace). The predictive effect survives in both designs; if this effect had been due to 'temporal smearing', applying a FIR filter on reversed time would eliminate the finding. Bottom row: Time domain zero-phase Butterworth (IIR) bandpass filter and Hilbert transform.

Supplementary Table 1: Cortical Stimulation Mapping
PT: planum temporale; HG: Heschl's gyrus; TTS: transverse temporal sulcus; PP: planum polare
AN: auditory-cued naming; VN: visually-cued naming; AR: auditory repetition

Patient 1

Contacts	mA	Location	Task	Result	Comments
A15-16	6	PT	AN	+	"I feel like there's other people talking." "There's something weird, somebody's talking, or there's stuff going on around me." "I'm feeling like there's something else going on over here (*gesturing to the right*). I almost feel like someone else was talking, other than you talking." (Like you're hearing something?) "Like I'm hearing someone else." (That only occurs when I stimulate you?) "Only when you stimulate." (You think you can hear through it?) "That's what I was trying to do."
A13-14	2	PT	AN	+	(What?) "People are feeling wicked … people talking … wishy-washy … wicky-wacky … what is that?" (Is it still there?) "People aren't talking like they were." (So what did you feel? Tell me about it.) "I felt – not shock like a pain, but a shock like a little vibrate. And then it felt like something was like 'what-cha-ma-call-it' was the word, and then kind of like … an echo, and then just kind of like a rolling of … more words, but nothing specific."
A11-12	2	PT			"There's people talking all around me."
A9-10	1	HG/TTS			(*gesturing to the right*) (But it's getting less intense?) "A little less intense, yeah, like walking in a mall right now. Before it was like a ballpark."
A7-8	6	HG	AR	+	
A7-8	6	HG	AN	+	
A7-8	6	HG	VN	-	
A8-9	2	HG	AR	+	"I hear something there."
A8-9	2	HG	AN	+	
A8-9	2	HG	VN	-	
A5-6	5	PP	AR	+	
A5-6	5	PP	AN	+	
A5-6	5	PP	VN	+	
A3-4	5	PP	AR	+	"Weird." (Why did you say weird? What happened?) "I heard what she said and then it just … devolved … just went away. Just like the words just crumbled away."
A3-4	5	PP	AN	+	
A3-4	5	PP	VN	-	
B9-10	5	pl-STG	AR	-	
B9-10	5	pl-STG	AN	-	
B9-10	5	pl-STG	VN	-	
C9-10	4	ml-STG	AR	-	
C9-10	4	ml-STG	AN	-	
C9-10	4	ml-STG	VN	-	
D9-10	4	al-STG	AR	-	
D9-10	4	al-STG	AN	-	
D9-10	4	al-STG	VN	-	

Patient 2

Contacts	mA	Location	Task	Result	Comments
A1-2	5	PP	AR	-	
A1-2	5	PP	AN	-	
A1-2	5	PP	VN	-	
A3-4	5	PP	AR	-	
A3-4	5	PP	AN	-	
A3-4	5	PP	VN	-	
A5-6	5	PP	AR	-	
A5-6	5	PP	AN	-	
A5-6	5	PP	VN	-	
A7-8	5	PP	AR	-	
A7-8	5	PP	AN	-	
A7-8	5	PP	VN	-	
A9-10	5	PP/HG	AR	+	
A9-10	5	PP/HG	AN	+	
A9-10	5	PP/HG	VN	-	
A10-11	4	HG			“Yeah, yeah. Did someone say something [incomprehensible] right now?” (Are you hearing something?) “Yeah. It’s kind of like a ringing in my ear.”
A10-11	4	HG	AR	+	“Kind of had a ringing in your ear. Not normal, something like …” (Does it affect your ability to hear [the question]?) “A little bit, or maybe putting the words together.”
A10-11	4	HG	VN	-	
A11-12	2	HG			“That was like a really bad echo I was hearing just now. It’s a really bad echo, almost like you’re talking in a cave or something.”
A11-12	2	HG	AR	+	“It’s [the echo] really bad.”
A11-12	2	HG	VN	-	
A12-13	1	HG/PT			“Yeah, that’s bad. The hearing on that is bad.” (Is it worse than before, the echo?) “It was up there. It’s very bad.”<br>(Still getting the echo?) “Really bad.”
A13-14	1	PT			“That’s really bad. It’s similar to the other one. A very quiet echo.” (A little different in frequency?) “Yes, lower.”<br>(What do you feel?) “It feels like you’re going underwater, when you’re underwater with the water in your ears.”
A14-15	1	PT			“That’s really bad. That’s similar to the previous one.”<br>“I can barely hear anything.”
A15-16	1	PT			“It’s a bad one.”
C9-10	5	ml-STG	AR	-	
C9-10	5	ml-STG	AN	-	
C9-10	5	ml-STG	VN	-	
D10-11	5	al-STG	AR	-	
D10-11	5	al-STG	AN	-	
D10-11	5	al-STG	VN	-	

Patient 3

Contacts	mA	Location	Task	Result	Comments
A1-2	7	PP	AR	-	
	7	PP	AN	-	
	7	PP	VN	-	
A4-5	7	PP	AR	+	
	7	PP	AN	+	
	7	PP	VN	-	
A6-7	7	HG	AR	+	
	7	HG	VN	-	
A8-9	5	HG	AR	-	
	5	HG	AN	-	
	5	HG	VN	-	
A10-11	3	HG/TTS			(*patient startles*) (Did you hear anything?) “I felt a shock or …” (Was it a shock or was it a sound?) “It was like a ‘brrrrr’, I can’t tell if it’s shocking me or what.”
	3	HG/TTS	AR	-	
	3	HG/TTS	AN	-	
	3	HG/TTS	VN	-	
A12-13	3	PT			(*patient startles*) (Was it the sound again? The buzzing again?) “Yeah. I could still hear you talking, though.” (You could still hear me talking?) “It’s like … it starts off with ‘brrrrr.’”
	3	PT	AR	-	(*speaking very loudly*)
	3	PT	AN	-	(*speaking very loudly*)
	3	PT	VN	-	(*speaking very loudly*)
A14-15	7	PT			(You hear anything?) “I can hear it.” (Is it as much as before?) “No, it wasn’t near as …” (Ok, good. How was that?) “I can still hear you talk.”
	7	PT	AR	+	(*speaking very loudly*)
	7	PT	AN	+	(*speaking very loudly*)
	7	PT	VN	+	(*speaking very loudly*)
B12-13	7	pl-STG	AR	+	
	7	pl-STG	AN	+	
	7	pl-STG	VN	+	
C11-12	7	ml-STG	AR	-	
	7	ml-STG	AN	-	
	7	ml-STG	VN	-	
D11-12	7	al-STG	AR	-	
	7	al-STG	AN	-	
	7	al-STG	VN	-	
