# Supplementary material for: Language prediction mechanisms in human auditory cortex
Source: Nat Commun. 2020 Oct 16;11:5240. doi: 10.1038/s41467-020-19010-6 (PMC7567874; doi:10.1038/s41467-020-19010-6)
Supplement: Supplementary file 2 — Reporting Summary [file 41467_2020_19010_MOESM2_ESM.pdf]

nature research

Corresponding author(s): Nitin Tandon

Reporting Summary

Nature Research wishes to improve the reproducibility of the work that we publish. This form provides structure for consistency and transparency in reporting. For further information on Nature Research policies, see Authors & Referees and the Editorial Policy Checklist.

Statistical parameters

When statistical analyses are reported, confirm that the following items are present in the relevant location (e.g. figure legend, table legend, main text, or Methods section).

n/a	Confirmed	
☐	☒	The exact sample size (*n*) for each experimental group/condition, given as a discrete number and unit of measurement
☐	☒	An indication of whether measurements were taken from distinct samples or whether the same sample was measured repeatedly
☐	☒	The statistical test(s) used AND whether they are one- or two-sided<br>*Only common tests should be described solely by name; describe more complex techniques in the Methods section.*
☒	☐	A description of all covariates tested
☐	☒	A description of any assumptions or corrections, such as tests of normality and adjustment for multiple comparisons
☐	☒	A full description of the statistics including central tendency (e.g. means) or other basic estimates (e.g. regression coefficient) AND variation (e.g. standard deviation) or associated estimates of uncertainty (e.g. confidence intervals)
☐	☒	For null hypothesis testing, the test statistic (e.g. *F*, *t*, *r*) with confidence intervals, effect sizes, degrees of freedom and *P* value noted<br>*Give P values as exact values whenever suitable.*
☒	☐	For Bayesian analysis, information on the choice of priors and Markov chain Monte Carlo settings
☒	☐	For hierarchical and complex designs, identification of the appropriate level for tests and full reporting of outcomes
☐	☒	Estimates of effect sizes (e.g. Cohen's *d*, Pearson's *r*), indicating how they were calculated
☐	☒	Clearly defined error bars<br>*State explicitly what error bars represent (e.g. SD, SE, CI)*

Our web collection on statistics for biologists may be useful.

Software and code

Policy information about availability of computer code

Data collection	Python v2.7<br>MATLAB R2014
Data analysis	MATLAB R2018b<br>R 3.4.0<br>AFNI/SUMA 17.2.00<br>Freesurfer 5.1.0

For manuscripts utilizing custom algorithms or software that are central to the research but not yet described in published literature, software must be made available to editors/reviewers upon request. We strongly encourage code deposition in a community repository (e.g. GitHub). See the Nature Research guidelines for submitting code & software for further information.

Data

Policy information about availability of data

All manuscripts must include a data availability statement. This statement should provide the following information, where applicable:

- Accession codes, unique identifiers, or web links for publicly available datasets
- A list of figures that have associated raw data
- A description of any restrictions on data availability

The individual datasets collected and analyzed during the current study are not publicly available as patients did not consent to such distribution, but all grouped data, their analyses and representations are available from the corresponding author on reasonable request.

Field-specific reporting

Please select the best fit for your research. If you are not sure, read the appropriate sections before making your selection.

☒ Life sciences ☐ Behavioural & social sciences ☐ Ecological, evolutionary & environmental sciences

For a reference copy of the document with all sections, see nature.com/authors/policies/ReportingSummary-flat.pdf

Life sciences study design

All studies must disclose on these points even when the disclosure is negative.

Sample size	More than 30 trials were collected for noise listening and spoken naming to definition in more than 30 patients.
Data exclusions	Exclusion criteria were pre-established and are standard for invasive electrophysiology in patients with epilepsy. 2 patients were excluded for gross structural brain anomalies (prior temporal lobe resections). 2 patients were excluded because the region of primary interest (auditory cortex) was involved in seizure onset. All other patients (n = 37) were included. All patients underwent the white noise task; due to clinical time constraints, a subset (n = 25) also completed the naming to definition task. In these patients, cortical areas with potentially abnormal physiology were excluded by removing channels that demonstrated inter-ictal activity or that recorded in proximity to the localized seizure onset sites. Additional channels contaminated by >10 dB of line noise or regular saturation were also excluded from further analysis. Any trials manifesting epileptiform activity were removed. Furthermore, trials for the naming to definition and reversed speech experiments in which the patient answered incorrectly or after more than 2 seconds were eliminated.
Replication	Each experiment involved multiple trials (technical replicates) in multiple patients (biological replicates). There were 100 trials of white noise and 78 trials of naming to definition; there were 37 patients. Each patient received the same instructions before each task, and no patient was exposed to the stimuli prior to the task. Both individual (technical variability) and group results (biological variability) are shown. Results are consistent within and across patients.
Randomization	White noise stimuli were produced uniquely for each trial with a random number generator seeded to the time of presentation. Naming to definition stimuli were presented in a random order. Patient were recruited in the order in which they were clinically admitted for intracranial monitoring.
Blinding	All patients were from the same cohort and consequently blinding was not applicable.

Reporting for specific materials, systems and methods

Materials & experimental systems

n/a	Involved in the study	
☒	☐	Unique biological materials
☒	☐	Antibodies
☒	☐	Eukaryotic cell lines
☒	☐	Palaeontology
☒	☐	Animals and other organisms
☐	☒	Human research participants

Methods

n/a	Involved in the study	
☒	☐	ChIP-seq
☒	☐	Flow cytometry
☐	☒	MRI-based neuroimaging

Human research participants

Policy information about studies involving human research participants

Population characteristics	37 patients with medically intractable epilepsy (mean age 33, 16 female, mean IQ 97) and no overt neurological deficits.
Recruitment	Patients with medically intractable epilepsy undergoing intracranial electrode implantation to localize seizure onset zones were enrolled in the study after written informed consent was obtained. Their participation in the study was voluntary, decoupled from their clinical care, and they were given the option of withdrawing participation at any time.

Magnetic resonance imaging

Experimental design

Design type	Pre-operative structural imaging
Design specifications	N/A
Behavioral performance measures	N/A

Acquisition

Imaging type(s)	Anatomical
Field strength	3T
Sequence & imaging parameters	Images were collected using a magnetization-prepared 180° radiofrequency pulse and rapid gradient-echo sequence with 1 mm sagittal slices and an in-plane resolution of 0.938 x 0.938 mm97.
Area of acquisition	Whole brain scan

Diffusion MRI ☐ Used ☒ Not used

Preprocessing

Preprocessing software	AFNI/SUMA 17.2.00<br>Freesurfer 5.1.0
Normalization	Linear 12-parameter affine transform
Normalization template	Colin_N27 in Talairach space
Noise and artifact removal	N
Volume censoring	None

Statistical modeling & inference

Model type and settings	N/A
Effect(s) tested	N/A

Specify type of analysis: ☒ Whole brain ☐ ROI-based ☐ Both

Statistic type for inference (See Eklund et al. 2016)	N/A
Correction	N/A

Models & analysis

n/a	Involved in the study
☒	☐ Functional and/or effective connectivity
☒	☐ Graph analysis
☒	☐ Multivariate modeling or predictive analysis
