# Supplementary material for: Language prediction mechanisms in human auditory cortex
Source: Nat Commun. 2020 Oct 16;11:5240. doi: 10.1038/s41467-020-19010-6 (PMC7567874; doi:10.1038/s41467-020-19010-6)
Supplement: Supplementary file 3 — Description of Additional Supplementary Files [file 41467_2020_19010_MOESM3_ESM.pdf]

Description of Additional Supplementary Files

File Name: Supplementary Movie 1
Description: **Response of the Supratemporal Plane to Rhythmic White Noise** Surface-based mixed-effects multilevel analysis of high-gamma power at all electrodes in superior temporal gyrus during listening to the 3 Hz amplitude-modulated white noise stimulus.

File Name: Supplementary Movie 2
Description: **Response of the Supratemporal Plane during Speech Perception** Surface-based mixed-effects multilevel analysis of high-gamma power at all electrodes in superior temporal gyrus during listening to natural language speech.

File Name: Supplementary Movie 3
Description: **Response of the Supratemporal Plane during Speech Production** Surface-based mixed-effects multilevel analysis of high-gamma power at all electrodes in superior temporal gyrus during single-word articulations

File Name: Supplementary Movie 4
Description: **Chronometric Stimulation of the Supratemporal Plane** The overt behavior of the first patient during chronometric cortical stimulation mapping with concurrent intracranial electrophysiology and precise localization of the stimulating electrodes relative to a patient-specific cortical model. The patient is evaluated with a sentence repetition task. Stimulation of HG/TTS during listening results in failure, but stimulation of HG/TTS during production does not alter behavior. In contrast, stimulation of PT during production results in failure.
